# Automated seed counting using image processing and deep learning

**DOI:** 10.3389/fpls.2025.1659781

**Published:** 2025-08-29

**Authors:** Qiuyu Zu, Teng Liu, Wenpeng Zhu, Yan Pan, Jinxu Wang, Xinru Song, Jialin Yu, Shu Dang, Xiaoming Yu, Zhenyu Zhang

**Affiliations:** ^1^ School of Agriculture, Jilin Agricultural Science and Technology College, Jilin City, China; ^2^ Peking University Institute of Advanced Agricultural Sciences, Shandong Laboratory of Advanced Agricultural Sciences in Weifang, Weifang, China

**Keywords:** image processing, deep learning, YOLOv5, mobile app, seed counting

## Abstract

**Introduction:**

Accurate seed counting is an essential task in agricultural research and farming, supporting activities such as crop breeding, yield prediction, and weed management. Traditional manual seed counting, while accurate, is time-consuming, labor-intensive, and prone to human error, particularly for large quantities of micro-sized seeds.

**Methods:**

This study developed two automated computer vision approaches integrated into a mobile application (app) for seed counting: one utilizing image processing (IP) and the other based on deep learning (DL). These methods aim to address the limitations of traditional manual counting by providing automated, efficient alternatives.

**Results:**

The IP-based method demonstrated high accuracy comparable to manual counting and offered substantial time savings. However, its reliance on controlled environmental conditions, such as uniform lighting, limits its versatility for field apps. The DL-based method excelled in speed and scalability, processing counts in as little as 0.33 seconds per image, but its accuracy was inconsistent for visually complex or densely clustered seeds.

**Discussion:**

Both automated methods significantly enhance the efficiency of seed counting, providing a practical and accessible solution for various agricultural contexts. The integration of these methods into a mobile app streamlines seed counting for laboratory research, field studies, seed production, and breeding trials, offering a transformative approach to modernizing seed counting practices while reducing time and labor requirements.

## 1 Introduction

Counting crop and weed seeds is a fundamental task in various agricultural research activities, playing a crucial role in advancing our understanding of plant sciences and optimizing agronomic practices (Zhao et al., 2018). In practical crop cultivation, variety control is generally performed from sowing through to the early vegetative growth stage to ensure uniform stand establishment and the expression of target traits (Folta, 2019). Precise seed counting in germination studies allows researchers to determine germination rates, assess seedling vigor, and evaluate the impact of different treatments or environmental conditions on seed viability (Devika et al., 2021). These insights are vital for developing effective cultivation practices and ensuring the successful establishment of crops (Colmer et al., 2020). Moreover, accurate seed counting is integral to crop yield estimation, aiding in predicting potential harvest outcomes and guiding resource allocation, such as water, fertilizers, and pesticides, to maximize productivity (Mgendi, 2024). In plant breeding programs, precise seed counting is crucial for selecting the best-performing varieties and monitoring their reproduction (Genze et al., 2020; Dhanya et al., 2024).

In weed management, counting weed seeds provides valuable insights into weed population dynamics and the effectiveness of control strategies (Liu et al., 2021; Kumar et al., 2024). By quantifying the seed bank in the soil, researchers can predict future weed infestations and develop targeted management practices to reduce weed pressure, thereby minimizing competition with crops (Schwartz-Lazaro and Copes, 2019). This practice is particularly significant in integrated weed management programs, which combine biological, mechanical, and chemical methods for sustainable weed control (Zhang et al., 2021). Furthermore, accurate seed counting is crucial for assessing germination rates and evaluating the impacts of environmental factors, such as light, soil moisture, and burial depth, on weed seed germination and seedling establishment (Marschner et al., 2024). The biological characteristics of seed germination obtained from these studies can be applied in integrated weed management practices, enhancing the sustainability and effectiveness of weed control strategies.

Traditionally, seed counting has been performed manually, a process that is time-consuming, labor-intensive, and prone to human error, particularly when dealing with large quantities of small seeds (Zhao et al., 2023). This highlights the necessity of developing automated methods to enhance the accuracy and efficiency of seed counting. As part of this research, image processing (IP) algorithms showed promise in accurately counting plant seeds. However, their performances were sensitive to variations in lighting, requiring controlled conditions, such as uniform lighting with an acrylic sheet background, for reliable results. This setup, however, is cumbersome and impractical for field applications.

Deep learning (DL) convolutional neural networks (CNNs) have emerged as powerful tools in agriculture, providing efficient solutions for counting plants, fruits, seeds, and other agricultural targets (Sa et al., 2016; Kamilaris and Prenafeta-Boldú, 2018b; Chen et al., 2020). By analyzing high-resolution images, CNNs can detect and quantify objects with remarkable accuracy, even under challenging conditions such as varying lighting, occlusions, and complex backgrounds (Redmon et al., 2016; Jin et al., 2024). This technology eliminates the need for manual counting, which is often labor-intensive, time-consuming, and prone to human error (Wen et al., 2022; Chen et al., 2024). Applications range from estimating crop yields and monitoring growth to evaluating seed germination rates and tracking pest infestations (Dong et al., 2021; Eron et al., 2024). By automating object counting, CNNs enable farmers and researchers to make data-driven decisions, optimize resource allocation, and enhance agricultural productivity, paving the way for smarter and more sustainable farming practices (Kamilaris and Prenafeta-Boldú, 2018a; Santos et al., 2020).

In our preliminary study, to overcome the limitations of using the IP-based method for counting seeds, we developed an innovative approach that utilizes DL CNNs for seed counting. This method integrates DL models into a mobile application (app), enabling seed counting by photographing seeds on simpler backgrounds, such as white A4 paper, orange or black table surfaces, thereby eliminating the use of acrylic sheets with controlled lighting. A significant innovation of this study is the integration of DL techniques, which enhances the robustness of seed counting under variable field conditions. Compared to traditional manual counting and previous image processing (IP) methods, this combined approach not only improves counting accuracy but also increases processing speed, scalability, and adaptability across diverse agricultural environments. Therefore, the primary objectives of this study were to compare and evaluate the accuracy and time efficiency of three seed counting methods: manual counting, IP-based seed counting, and DL-based counting, aiming to determine which method provides the most reliable and efficient solution for counting crop and weed seeds. The comparison will offer valuable insights into the strengths and limitations of each method, guiding future apps and research in automated seed counting.

## 2 Materials and methods

### 2.1 Seed selection

This study offered a comprehensive evaluation of the accuracy and time efficiency of three seed counting methods: manual counting, IP-based counting, and DL-based counting. The primary objective was to compare these methods to determine their relative accuracy and efficiency. To ensure a comprehensive evaluation, we selected a diverse range of crop and weed seeds, representing various sizes, shapes, and colors, including turfgrasses, row crops, and weeds. A total of 15 different plant seeds, including alfalfa, barnyardgrass, common beggarticks, common cocklebur, goosegrass, Kentucky bluegrass, maize, musk mallow, peanut, perilla mint, redroot pigweed, smooth pigweed, velvetleaf, wheat, and zoysiagrass, were tested, as summarized in **Table 1**.

**Table 1:** Plant species and their seeds used in the study.

Common Name	Scientific Name
Alfalfa	*Medicago sativa* L.
Barnyardgrass	*Echinochloa crus-galli* (L.) P. Beauv.
Common beggarticks	*Bidens pilosa* L.
Common cocklebur	*Xanthium strumarium* L.
Goosegrass	*Eleusine indica* (L.) Gaertn.
Kentucky bluegrass	*Poa pratensis* L.
Maize	*Zea mays* L.
Musk mallow	*Malva moschata* L.
Peanut	*Arachis hypogaea* L.
Perilla mint	*Perilla frutescens* (L.) Britton
Redroot pigweed	*Amaranthus retroflexus* L.
Smooth pigweed	*Amaranthus hybridus* L.
Velvetleaf	*Abutilon theophrasti* Medik.
Wheat	*Triticum aestivum* L.
Zoysiagrass	*Zoysia japonica* Steud.

In this study, two methods were implemented for seed counting: an IP-based method and a DL-based method. The IP-based method was deployed on the cloud to take advantage of its scalability and centralized processing capabilities. This allows for consistent performance across different devices and simplifies updates, ensuring that the method remains effective regardless of hardware or operating system variations. In comparison, the DL method was implemented locally on mobile devices, aiming to explore the trade-off between resource requirements and real-time performance.

### 2.2 IP-based counting methods and mobile app development

**Figure 1:**
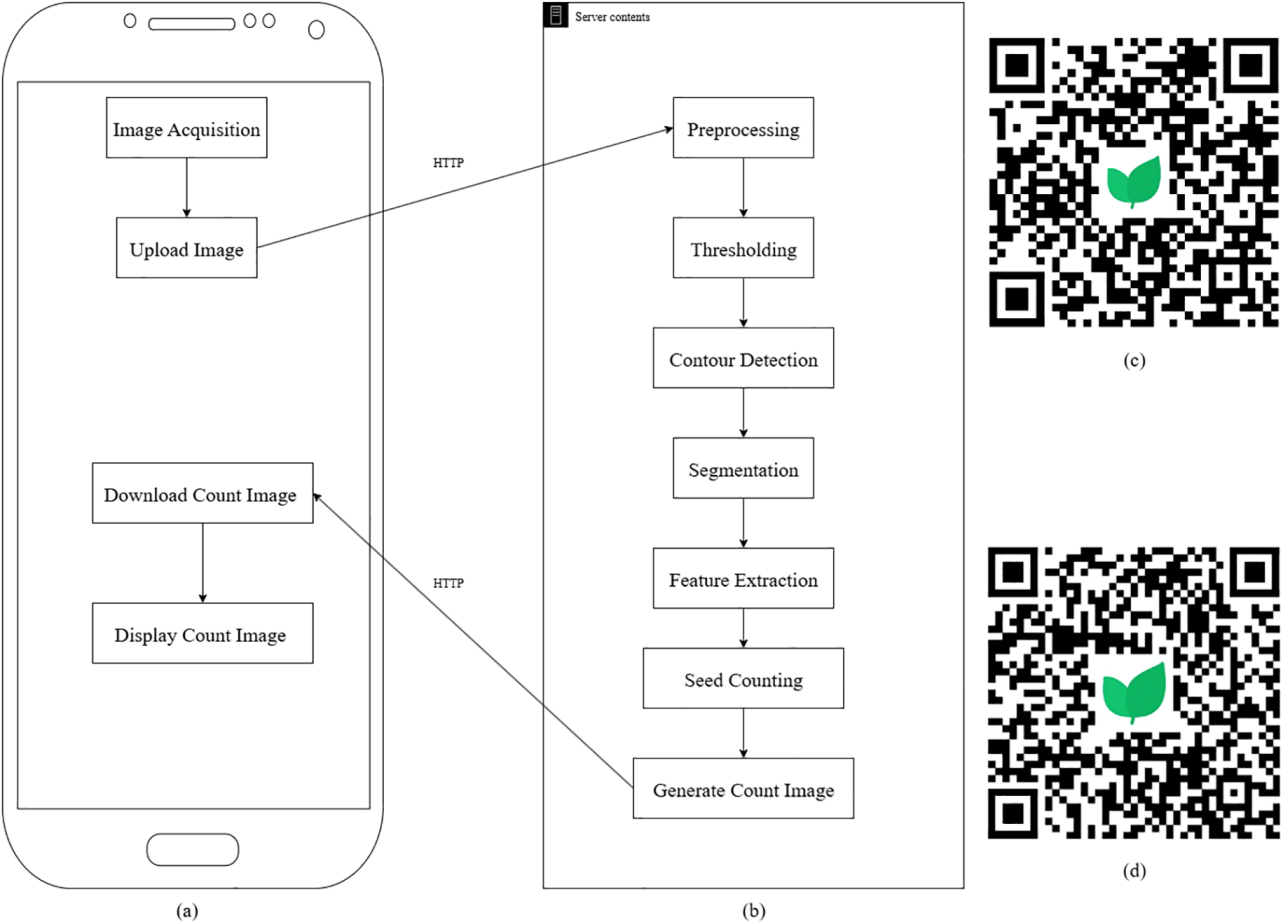
Image processing (IP)-based flowchart: **(a)** application (app) flowchart, **(b)** server flowchart, **(c)** Quick Response (QR) code for downloading the Android app, **(d)** QR code for downloading the iPhone Operating System app (iOS).

The IP algorithm was developed using custom-written code to detect and count seeds from images. The workflow, illustrated in **Figure 1**, involves a series of IP steps. Key steps include:

1. Image acquisition:
The first step in the IP-based seed counting method involves capturing high-resolution images of the seed samples using a smartphone camera. The images are captured under controlled lighting conditions to minimize shadows and ensure uniform illumination, ensuring consistent image quality across different seed samples.
2. Preprocessing:
The acquired image is first converted to grayscale, simplifying the image by removing color information and focusing on intensity variations. A Gaussian blur is applied to the grayscale image to smooth it and reduce noise, which helps improve the quality of the subsequent steps, such as thresholding and segmentation.
3. Thresholding:
Binary thresholding is applied to the blurred grayscale image. This step converts the image into a binary format, where pixel values above a certain threshold are set to white (foreground), and the rest are turned black (background). This simplifies the task of distinguishing the seeds from the background, enabling easier detection of regions of interest.
4. Contour detection:
This step involves detecting the contours of the binary image using the cv2.findContours() function. We applied the external contour retrieval mode (cv2.RETR_EXTERNAL) to ensure only the outermost contours of the detected objects (seeds) are considered. Additionally, the simple chain approximation method (cv2.CHAIN_APPROX_SIMPLE) is used to simplify the contour representation by storing only the endpoints of straight-line segments, thereby reducing computational overhead. These choices optimize both the efficiency and accuracy of contour detection, enabling the precise identification of seed boundaries.
5. Segmentation:
After contour detection, the image is segmented by identifying connected components. Each connected region represents a potential seed. These regions are sorted based on their y-coordinate to ensure spatial organization, maintaining the relative positioning of seeds in the original image.
6. Feature extraction:
For each segmented region, key features such as bounding rectangles, dimensions (length and width), and aspect ratio (L/W) are calculated. These features are then converted from pixels to real-world measurements (millimeters) using a reference object or scale, ensuring that the counting is accurate and meaningful.
7. Seed counting:
Once the contours and features have been extracted, the number of connected components or distinct regions is counted. Each seed is labeled, and a unique identifier is assigned, corresponding to its position in the image. This number is the final seed count, providing a reliable estimate based on the detected regions.

Due to the limitations of the mobile device, the algorithm was deployed to the cloud. This cloud environment enabled dynamic scaling of resources, allowing for the execution of complex IP algorithms without the need for expensive, on-premises infrastructure. This flexibility improved the handling of large datasets and processing-intensive tasks, such as real-time image recognition (Dritsas and Trigka, 2025). Automating the deployment process in the cloud reduced the time required for deploying IP-based apps by eliminating manual configuration and ensuring consistent environments across multiple nodes (Etchevers et al., 2017). Cloud deployment offered cost savings by utilizing shared resources and reducing the need for dedicated hardware. The pay-as-you-go model in cloud computing minimizes expenses while allowing the efficient execution of IP-based apps (Xia et al., 2021).

The algorithm was integrated into the mobile app PhenoTyper^®^, available on iOS and Android platforms, enabling users to capture photos of seeds for automatic seed counting (**Figure 1**). This method utilized an acrylic sheet with lighting to ensure optimal image quality.

High-resolution images of these seed samples were captured using a smartphone (Huawei Maimang 11, Huawei Technologies Co., Ltd., Shenzhen, China). The seeds were placed on a uniform, non-reflective surface, and controlled lighting was provided by an acrylic sheet to minimize shadows and ensure consistent illumination across all samples. The IP-based counting methods were implemented within the app, which was developed explicitly for this study to streamline the seed counting process.

The mobile app first accessed the device’s photo album or camera to acquire images of seeds based on the user’s input. Once captured or selected, these images were uploaded to a cloud-based server via the Hypertext Transfer Protocol (HTTP) for further analysis. On the cloud server, an advanced image recognition algorithm processed the images to accurately count the seeds. Following the analysis, the server returned the seed count results to the app, which then displayed them to the user. By offloading computationally intensive image recognition tasks to the cloud, this approach ensured rapid and efficient seed count feedback, significantly enhancing the app’s performance and user experience.

### 2.3 DL-based counting models and mobile app development

#### 2.3.1 Training and testing

The high-resolution images for the DL-based counting models were captured using the same device as the IP-based methods, with the background being white A4 paper and the surface being orange or black table surfaces. The images were annotated to create bounding boxes around each seed using LabelImg, an opensource graphical image annotation tool developed by Tzutalin (2015) and available on GitHub (Tzutalin, 2015; https://github.com/tzutalin/labelImg). The annotated data were then split into training, validation, and testing sets, with a ratio of 8:1:1 to ensure robust model evaluation. Although the 8:1:1 data split provides a reasonable starting point, future studies may consider alternative evaluation strategies such as larger test sets or cross-validation.

- Background: White A4 paper.
- Surface: Orange or black table surface.
- Lighting: Standard indoor lighting.

Photos of various seeds were taken under consistent conditions at a resolution of 3024 × 4032 pixels. These images were then labeled manually to indicate the exact location and count of seeds. A total of 900 images were captured, with 300 images taken on a standard white A4 paper background, 300 images on an orange table surface, and 300 images on a black table surface. The number of seeds in each image varied across the dataset to reflect practical counting scenarios. Images were prepared to include a wide range of seed densities, and the distribution was randomized to ensure model generalization across different seed quantities. To better simulate real-world conditions, expanding the dataset to include images under various lighting conditions, varying seed sizes, and types would be beneficial for increasing model generalization. Future work will focus on collecting more diverse images in varied environments to enhance model performance and reliability across different scenarios.

The You Only Look Once (YOLO) model, specifically YOLOv5, was selected for training due to its ease of deployment on Android devices. Training was conducted using a workstation equipped with a GeForce RTX 3080 Ti GPU and 64 GB of memory to accelerate the computation process. For optimization, we employed several techniques, including data augmentation methods such as rotation, scaling, and flipping. These techniques helped enhance model robustness and generalization. To further enhance model performance and ensure more efficient training, additional optimization strategies, such as learning rate schedules, regularization methods, and early stopping, will be explored. To maintain consistency across models, the following hyperparameters were uniformly applied in each experimental setup:

- Training epochs: 200
- Learning rate: 0.001
- Batch size: 16
- Momentum: 0.937
- Weight decay: 0.0005

During the training and testing phases, the performance of the YOLOv5 model was evaluated using the following metrics.

Mean Average Precision (mAP) evaluates a model’s ability to detect and localize objects across multiple classes. It combines precision and recall by calculating the Average Precision (AP) for each class and averaging them. The formula is expressed as follows (Equation 1), where (N) is the number of classes and (AP_i_) is the AP for class (i):


(1) $$mAP=\frac{1}{N}\sum_{i=1}^{N}AP_{i}$$

Intersection over Union (IoU) measures the overlap between a predicted bounding box and the ground truth box. It is calculated as the ratio of the area of overlap to the area of union, calculated as shown in (Equation 2):


(2) $$IoU=\frac{Area\;of\;Overlap}{Area\;of\;Union}$$

Precision is the ratio of true positives to the total number of predicted positives (true positives + false positives) as illustrated in (Equation 3):


(3) $$Precision=\frac{True\;Positives}{True\;Positives+False\;Positives}$$

Recall, as defined in (Equation 4), is the ratio of true positives to the total number of actual positives (true positives + false negatives):


(4) $$Recall=\frac{True\;Positives}{True\;Positives+False\;Negatives}$$

F1 score, as shown in (Equation 5), is the harmonic mean of precision and recall, providing a balance between the two:


(5) $$F1\;Score=\frac{2\times Precision\times Recall}{Precision+Recall}$$

#### 2.3.2 Deployment

The final models were evaluated on the test set to assess their performance in a real-world scenario. mAP, IoU, precision, recall, and F1 score were calculated to measure the model’s effectiveness. The model’s seed counts were compared to the manually counted ground truth to assess accuracy and time efficiency.

The model was integrated into the mobile app, enabling users to take photos and automatically count seeds against a background of A4 paper, orange or black table surface.

For the deployment of the model on Android, this study employed the TensorFlow Lite platform, a lightweight version of Google’s TensorFlow, specifically designed for mobile and embedded device platforms (Deng, 2019). TensorFlow Lite enables on-device machine learning, reducing dependency on cloud services and facilitating real-time data processing and inference, which was critical for apps requiring low latency or offline operation (Jouini et al., 2024).

Optimized for platforms with limited computational and memory resources, TensorFlow Lite employs techniques such as quantization and model optimization to efficiently execute models on devices with constrained power and memory, making it ideal for TinyML apps (Robert et al., 2021). Its capability to perform real-time inference on edge devices, such as smartphones and IoT devices, enhances decision-making speed by eliminating the need for cloud-based processing (Dai, 2020). TensorFlow Lite supported a wide range of mobile and embedded platforms, allowing developers to deploy machine learning models across various devices with minimal configuration. Its compact size and optimization for Advanced RISC Machine (ARM) architectures made it especially well-suited for mobile and embedded systems (Zeroual et al., 2019).

During model inference, hardware resource consumption was monitored using Android Profiler (Android Studio Narwhal) on a low-end Xiaomi Redmi Note 13 (8GB RAM, MediaTek Dimensity 6080). Results showed that the model required approximately 350MB of RAM and less than 15% CPU utilization per inference, demonstrating efficient resource management. While these results indicate that the model can efficiently run on low-end devices, as it is trained on larger datasets or becomes more complex (e.g., incorporating additional seed species or diverse backgrounds), memory usage and computational load are expected to increase. Larger datasets will require more storage space for model weights and preprocessing, which could potentially impact real-time performance, especially on resource-constrained devices. Future work will focus on optimizing model parameters, evaluating performance on a broader range of devices, and employing techniques such as quantization and model pruning to reduce model size, thereby improving its scalability for real-world applications.

The mobile app was used to acquire images, either by accessing the photo gallery or using the device’s camera function. Once the image was obtained, it underwent a preprocessing phase, during which the image was resized and adjusted to match the specific input dimensions required by the detection model. This preprocessing step ensured that the input data conformed to the model’s requirements, enabling optimal performance during detection.

**Figure 2:**
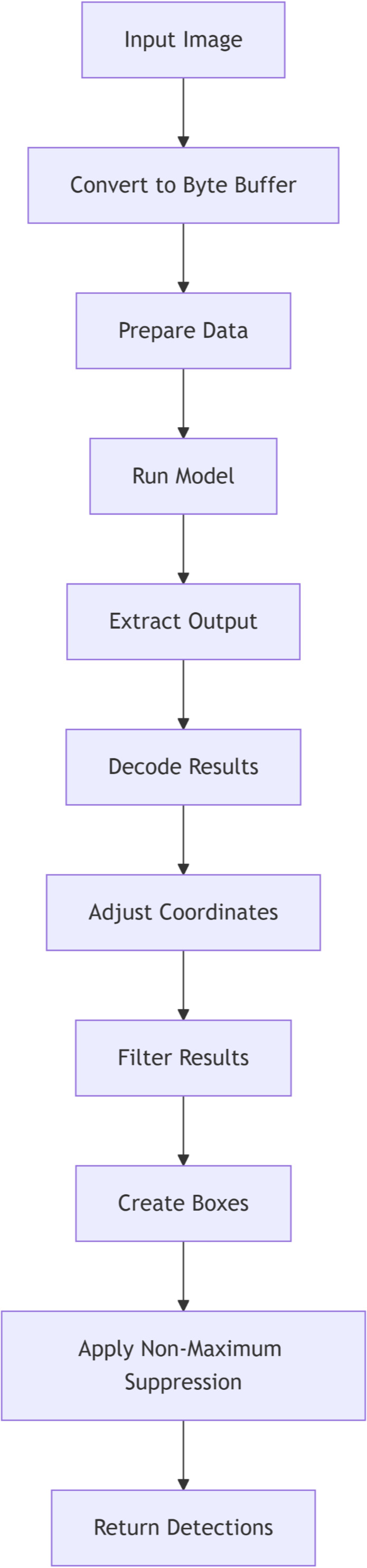
Flowchart illustrating the deep learning (DL)-based seed counting method.

After the preprocessing stage, the detection model was invoked to analyze the processed image. The model performed object detection based on pre-trained parameters, identifying relevant features within the image. The primary output of this process was the seed count, derived through image analysis algorithms integrated into the model. The final seed count results were recorded and stored for subsequent analysis. The DL-based flowchart is illustrated in **Figure 2**. Key steps included:

- Input image: Accepts a bitmap image as the starting point for the detection process.
- Convert to byte buffer: Converts the input image into a byte buffer suitable for feeding into the TensorFlow Lite model.
- Prepare data: Prepares input tensors and an output map to hold the predictions generated by the model.
- Run model: Executes inference using the TensorFlow Lite model with the input data.
- Extract output: Retrieves raw detection results from the model’s output map in the form of a byte buffer.
- Decode results: Processes the raw output by iterating through bounding boxes and extracting confidence scores and class probabilities.
- Adjust coordinates: Converts normalized box coordinates from the model into actual pixel dimensions relative to the input image size.
- Filter results: Filters detections by retaining only those with a class confidence greater than the specified threshold.
- Create boxes: Generates rectangle bounding box objects to represent the detected regions in the original image space.
- Apply non-maximum suppression: Performs Non-Maximum Suppression to reduce redundant overlapping boxes, keeping the most relevant detections.
- Return detections: Outputs a finalized list of detections as recognition objects, each containing labels and bounding box details.

### 2.4 Seed counting accuracy and time efficiency measurements

For each species, a total of 10, 70, and 100 seeds were manually counted by experienced personnel to establish a ground truth for comparison among the manual counting, IP-based counting, and DL-based counting methods. These quantities represent a range of seed counts reflecting both small- and larger-scale scenarios typical in real-world applications. The count of 10 seeds simulates small, easily manageable batches, while 70 and 100 seeds represent more common agricultural situations where seed quantities vary but remain within human counting capacity. This variation allowed assessment of each method’s accuracy and time efficiency across different counting scales.

During the manual counting phase, six human counters independently counted the seeds, with the time taken and the results recorded for each count. The mobile app, equipped with IP methods, was used to automatically count seeds in the images, with both processing time and resulting seed count recorded. Similarly, the mobile app with DL models, specifically YOLOv5, was employed to count the seeds in the images, and its processing time and resulting seed count were also recorded.

Time estimates were performed to compare the computational efficiency of the two methods within their deployment environments. The IP-based method was deployed on the cloud, leveraging scalable resources for large datasets, while the DL-based method was implemented locally on mobile devices to test its real-time performance. This comparison enabled evaluation of the efficiency trade-offs between cloud scalability and on-device processing.

To ensure fair and consistent evaluation of time efficiency across the three methods, the measurement of processing time was standardized. For manual counting, the duration was recorded from the moment the operator began inspecting the seeds until the final count was written down. For the IP-based method, time was measured from when the image was uploaded to the cloud server to when the seed count result was returned to the app. For the DL-based method, timing began when the image was loaded into the mobile device’s detection model and ended once the final count was produced. In all cases, the time measurements excluded image acquisition (e.g., taking or selecting photos) and user interactions, focusing solely on the core processing steps involved in obtaining the seed count.

### 2.5 Statistical analysis

Statistical analysis was performed to compare the accuracy and time efficiency between the three methods: manual counting, IP-based counting, and DL-based counting. Analysis of variance (ANOVA) was performed in SAS (SAS Institute, Cary, NC, United States). Data were checked for normality and homogeneity of equal variances prior to analysis. The data collection included a comprehensive set of metrics for each image, allowing for a thorough evaluation of each counting method. The results were summarized in terms of accuracy and time efficiency, with treatment means ± standard errors to compare the performance of each method. Treatment means were compared with Fisher’s Protected LSD at the 0.05 significance level.

## 3 Results

### 3.1 Model performance

**Table 2** summarizes the model’s performance across several key evaluation metrics. Precision was 0.963, indicating that 96.3% of the predicted positive instances were correct. Recall was 0.911, meaning the model detected 91.1% of the actual positive instances. The F1 Score, which balances both precision and recall, was 0.936, reflecting a solid trade-off between these metrics. The model also achieved a high mAP50 of 0.951, demonstrating its ability to accurately localize objects with an IoU threshold of 0.5. These results highlight the model’s high detection accuracy and robust localization capabilities.

**Table 2:** Model performance metrics.

Metric	Value
mAP50	0.951
Precision	0.963
Recall	0.911
F1 Score	0.936

### 3.2 Seed count result

**Figure 3:**
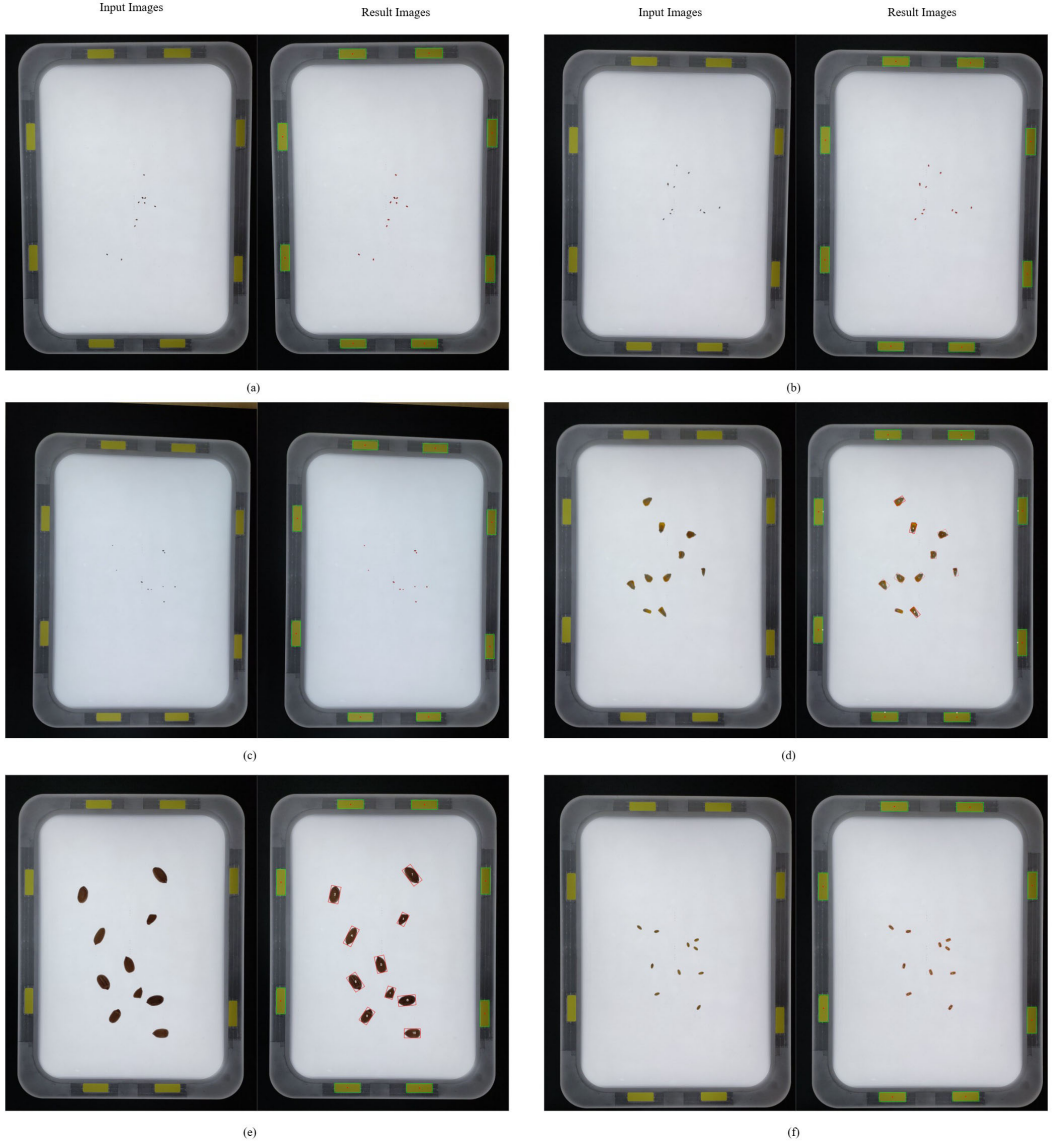
IP-based seed counting: **(a)** alfalfa, **(b)** barnyardgrass, **(c)** goosegrass, **(d)** maize, **(e)** peanut, **(f)** wheat.

**Figure 4:**
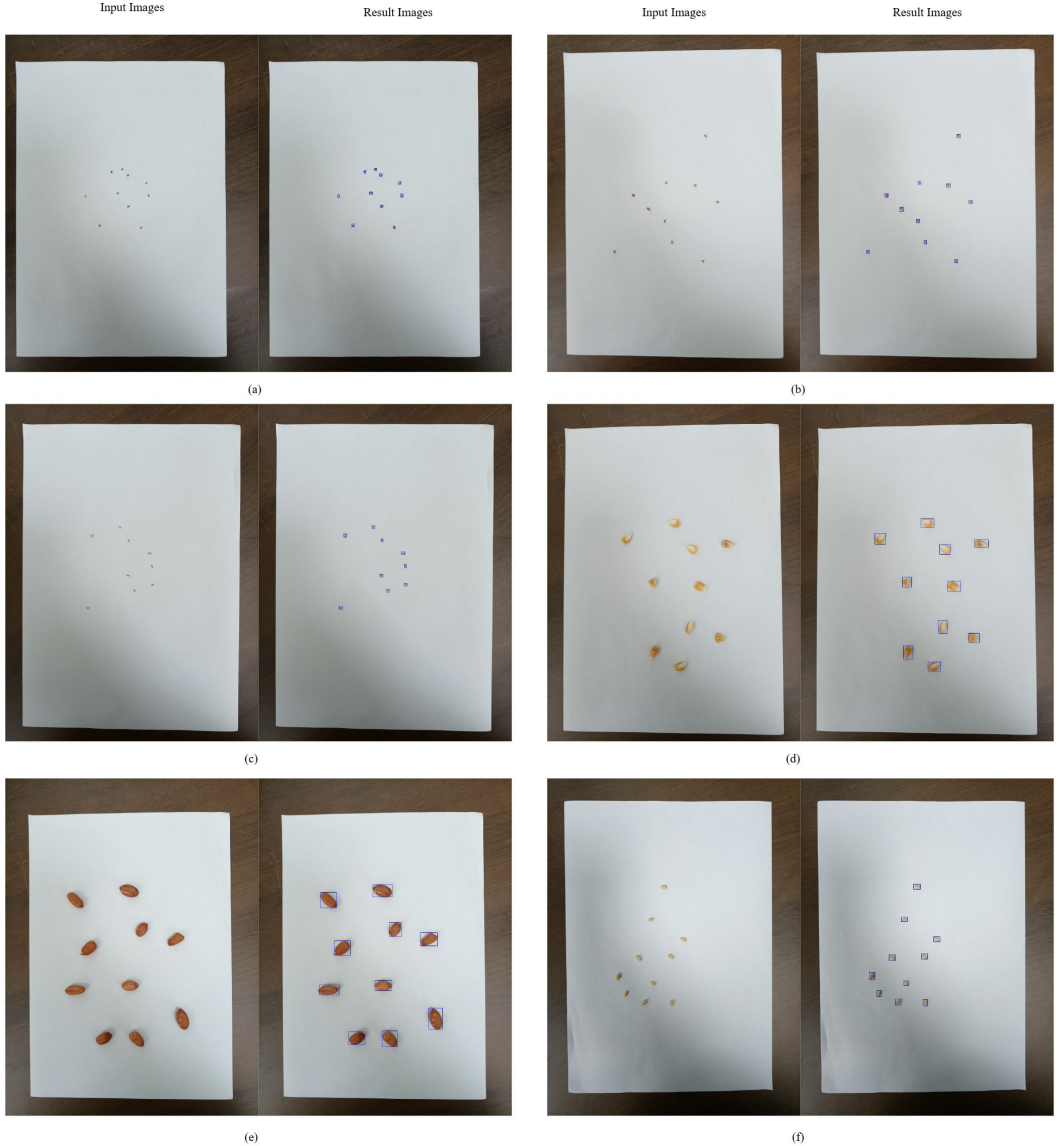
DL-based seed counting: **(a)** alfalfa, **(b)** barnyardgrass, **(c)** goosegrass, **(d)** maize, **(e)** peanut, **(f)** wheat.

**Figure 3** illustrates the counting results obtained using the IP-based method, while **Figure 4** presents the counting results derived from the DL-based approach. As summarized in **Table 3**, manual counting consistently achieved excellent accuracy across all tested species and seed quantities, with no deviations observed (e.g., 10.0 ± 0.0, 70.0 ± 0.0, 100.0 ± 0.0 for every species). This method served as the baseline for evaluating the precision of automated counting methods.

**Table 3:** Seed counting accuracy^a^.

Plant species	Methods^c^	Ground truth seed count^b^		
		10 seeds	70 seeds	100 seeds
		——————–Seed count———————		
Alfalfa	Manual	10.0 ± 0.0	70.0 ± 0.0	100.0 ± 0.0
	Image processing	10.0 ± 0.0	70.17 ± 0.17	100.0 ± 0.0
	Deep learning	10.0 ± 0.0	70.0 ± 0.0	100.0 ± 0.0
	p-value	NS	NS	NS
Barnyardgrass	Manual	10.0 ± 0.0	70.0 ± 0.0	100.0 ± 0.0
	Image processing	10.17 ± 0.17	70.67 ± 0.33	100.5 ± 0.22
	Deep learning	10.0 ± 0.0	70.0 ± 0.0	100.0 ± 0.0
	p-value	NS	NS	NS
Common beggarticks	Manual	10.0 ± 0.0	70.0 ± 0.0	100.0 ± 0.0
	Image processing	10.0 ± 0.0	70.0 ± 0.0	100.0 ± 0.0
	Deep learning	10.0 ± 0.0	70.0 ± 0.0	100.0 ± 0.0
	p-value	NS	NS	NS
Common cocklebur	Manual	10.0 ± 0.0	70.0 ± 0.0	100.0 ± 0.0
	Image processing	10.0 ± 0.0	70.0 ± 0.0	100.0 ± 0.0
	Deep learning	10.0 ± 0.0	70.0 ± 0.0	100.0 ± 0.0
	p-value	NS	NS	NS
Goosegrass	Manual	10.0 ± 0.0	70.0 ± 0.0	100.0 ± 0.0
	Image processing	10.33 ± 0.33	70.0 ± 0.0	100.0 ± 0.26
	Deep learning	10.0 ± 0.0	70.0 ± 0.0	99.83 ± 0.17
	p-value	NS	NS	NS
Kentucky bluegrass	Manual	10.0 ± 0.0	70.0 ± 0.0 a	100.0 ± 0.0 a
	Image processing	10.0 ± 0.0	70.0 ± 0.0 a	100.33 ± 0.21 a
	Deep learning	10.0 ± 0.0	39.83 ± 0.54 b	86.0 ± 0.0 b
	p-value	NS	***	***
Maize	Manual	10.0 ± 0.0	70.0 ± 0.0	100.0 ± 0.0
	Image processing	10.33 ± 0.21	70.0 ± 0.0	100.0 ± 0.0
	Deep learning	10.0 ± 0.0	70.0 ± 0.0	100.0 ± 0.0
	p-value	NS	NS	NS
Musk mallow	Manual	10.0 ± 0.0	70.0 ± 0.0	100.0 ± 0.0
	Image processing	10.0 ± 0.0	70.17 ± 0.17	100.33 ± 0.21
	Deep learning	10.0 ± 0.0	70.0 ± 0.0	100.0 ± 0.0
	p-value	NS	NS	NS
Peanut	Manual	10.0 ± 0.0	70.0 ± 0.0	100.0 ± 0.0
	Image processing	10.0 ± 0.0	70.0 ± 0.0	100.0 ± 0.0
	Deep learning	10.0 ± 0.0	70.0 ± 0.0	100.0 ± 0.0
	p-value	NS	NS	NS
Perilla mint	Manual	10.0 ± 0.0	70.0 ± 0.0	100.0 ± 0.0
	Image processing	10.0 ± 0.0	70.0 ± 0.0	100.0 ± 0.0
	Deep learning	10.0 ± 0.0	70.0 ± 0.0	100.0 ± 0.0
	p-value	NS	NS	NS
Redroot pigweed	Manual	10.0 ± 0.0 a	70.0 ± 0.0 a	100.0 ± 0.0 a
	Image processing	10.0 ± 0.0 a	70.0 ± 0.0 a	100.0 ± 0.0 a
	Deep learning	1.17 ± 0.31 b	18.17 ± 0.17 b	23.33 ± 0.71 b
	p-value	***	***	***
Smooth pigweed	Manual	10.0 ± 0.0 a	70.0 ± 0.0 a	100.0 ± 0.0 a
	Image processing	10.0 ± 0.0 a	70.0 ± 0.0 a	100.0 ± 0.0 a
	Deep learning	5.0 ± 0.26 b	35.17 ± 0.17 b	27.33 ± 0.42 b
	p-value	***	***	***
Velvetleaf	Manual	10.0 ± 0.0	70.0 ± 0.0	100.0 ± 0.0
	Image processing	10.0 ± 0.0	70.0 ± 0.0	100.0 ± 0.0
	Deep learning	10.0 ± 0.0	70.0 ± 0.0	100.0 ± 0.0
	p-value	NS	NS	NS
Wheat	Manual	10.0 ± 0.0	70.0 ± 0.0	100.0 ± 0.0
	Image processing	10.0 ± 0.0	70.0 ± 0.0	100.0 ± 0.0
	Deep learning	10.0 ± 0.0	70.0 ± 0.0	100.0 ± 0.0
	p-value	NS	NS	NS
Zoysiagrass	Manual	10.0 ± 0.0	70.0 ± 0.0	100.0 ± 0.0
	Image processing	10.0 ± 0.0	70.0 ± 0.0	100.0 ± 0.0
	Deep learning	10.0 ± 0.0	70.0 ± 0.0	100.0 ± 0.0
	p-value	NS	NS	NS

a NS, not significant; ***significant at the p-value ≤ 0.001 level.

b Ground truth seed count was manually verified by experienced personals used for accuracy comparison.

c For the same plant species, means within the column followed by the same letter are not significantly different based on Fisher’s Protected LSD at the 0.05 probability level.

The IP-based counting method demonstrated high accuracy for most species and seed counts, although minor deviations were observed in a few instances. For example, it counted 70 seeds of barnyardgrass with a mean of 70.67 and a standard error (SE) of ± 0.33 and 100 seeds with a mean of 100.5 and an SE of ± 0.22. Despite these small discrepancies, IP-based counting generally maintained a high level of precision comparable to manual counting. Statistical analysis revealed no significant differences (p > 0.05) between manual and IP-based counts for most species.

DL-based counting was effective for several species but demonstrated notable undercounting in specific cases, particularly for higher seed quantities in certain species. For example, the model counted only 27.33 (± 0.42) seeds out of 100 for smooth pigweed and 39.83 (± 0.54) seeds out of 70 for Kentucky bluegrass, both representing significant undercounts (p< 0.05) compared to manual and IP-based methods. Similar issues were observed in redroot pigweed, where only 23.33 (± 0.71) seeds out of 100 were counted. These findings indicate that while DL may be viable for certain species, its accuracy varies significantly depending on the specific seed quantity and types, specifically for higher counts or smaller, more visually challenging seed types, where it underperformed.

### 3.3 Time (s) consumed for seed counting

As shown in **Table 4**, the time required for seed counting varied significantly among the three methods. Manual counting was the most time-consuming, with time requirements increasing markedly as seed counts grew. For instance, counting 100 seeds of maize manually required 76.1 (± 1.24) seconds, while 100 seeds of smooth pigweed took 93.59 (± 1.84) seconds. This illustrates the limited scalability of manual counting for high-throughput apps, as it becomes progressively slower with larger seed quantities.

**Table 4:** Time efficiency for seed counting^a^.

Plant species	Methods^c^	Ground truth seed count^b^		
		10 seeds	70 seeds	100 seeds
		——————–Seconds———————		
Alfalfa	Manual	4.09 ± 0.12 a	30.75 ± 1.55 a	48.11 ± 3.07 a
	Image processing	3.78 ± 0.35 a	4.43 ± 0.33 b	2.8 ± 0.23 b
	Deep learning	0.32 ± 0.0 b	0.33 ± 0.01 c	0.33 ± 0.0 c
	p-value	***	***	***
Barnyardgrass	Manual	4.65 ± 0.04 a	39.59 ± 1.27 a	68.36 ± 1.36 a
	Image processing	2.96 ± 0.19 b	4.0 ± 0.21 b	5.92 ± 0.42 b
	Deep learning	0.33 ± 0.0 c	0.35 ± 0.0 c	0.35 ± 0.0 c
	p-value	***	***	***
Common beggarticks	Manual	5.38 ± 0.13 a	33.99 ± 1.08 a	54.41 ± 1.41 a
	Image processing	4.12 ± 0.44 b	3.81 ± 0.2 b	4.75 ± 0.35 b
	Deep learning	0.33 ± 0.0 c	0.33 ± 0.0 c	0.36 ± 0.0 c
	p-value	***	***	***
Common cocklebur	Manual	4.35 ± 0.14 a	32.85 ± 0.81 a	56.94 ± 3.01 a
	Image processing	3.83 ± 0.24 b	2.09 ± 0.48 b	4.31 ± 0.46 b
	Deep learning	0.33 ± 0.0 c	0.35 ± 0.0 c	0.36 ± 0.0 c
	p-value	***	***	***
Goosegrass	Manual	6.23 ± 0.42 a	21.33 ± 2.04 a	50.51 ± 2.24 a
	Image processing	5.77 ± 0.43 b	3.99 ± 0.27 b	3.66 ± 0.32 b
	Deep learning	0.33 ± 0.0 c	0.33 ± 0.0 c	0.34 ± 0.0 c
	p-value	***	***	***
Kentucky bluegrass	Manual	3.82 ± 0.41 a	48.42 ± 2.2 a	67.22 ± 1.49 a
	Image processing	2.35 ± 0.15 b	5.45 ± 0.37 b	2.9 ± 0.42 b
	Deep learning	0.33 ± 0.0 c	0.33 ± 0.0 c	0.33 ± 0.0 c
	p-value	***	***	***
Maize	Manual	4.68 ± 0.43 a	43.81 ± 1.8 a	76.1 ± 1.24 a
	Image processing	4.72 ± 0.21 a	4.04 ± 0.22 b	3.69 ± 0.34 b
	Deep learning	0.33 ± 0.0 b	0.35 ± 0.0 c	0.38 ± 0.0 c
	p-value	***	***	***
Musk mallow	Manual	4.49 ± 0.27 a	34.8 ± 2.84 a	55.18 ± 0.83 a
	Image processing	4.35 ± 0.22 a	3.59 ± 0.4 b	4.17 ± 0.35 b
	Deep learning	0.34 ± 0.0 b	0.34 ± 0.0 c	0.34 ± 0.0 c
	p-value	***	***	***
Peanut	Manual	5.13 ± 0.07 a	51.15 ± 2.43 a	51.84 ± 0.86 a
	Image processing	2.37 ± 0.39 b	5.68 ± 0.3 b	4.71 ± 0.21 b
	Deep learning	0.33 ± 0.0 c	0.36 ± 0.0 c	0.38 ± 0.0 c
	p-value	***	***	***
Perilla mint	Manual	4.99 ± 0.14 a	45.77 ± 1.73 a	70.74 ± 2.06 a
	Image processing	2.63 ± 0.28 b	5.46 ± 0.29 b	3.7 ± 0.47 b
	Deep learning	0.33 ± 0.0 c	0.33 ± 0.0 c	0.34 ± 0.0 c
	p-value	***	***	***
Redroot pigweed	Manual	4.04 ± 0.22 a	42.15 ± 3.21 a	71.13 ± 1.57 a
	Image processing	3.16 ± 0.4 b	4.62 ± 0.25 b	4.52 ± 0.31 b
	Deep learning	0.33 ± 0.0 c	0.33 ± 0.0 c	0.33 ± 0.0 c
	p-value	***	***	***
Smooth pigweed	Manual	4.52 ± 0.27 a	36.63 ± 1.58 a	93.59 ± 1.84 a
	Image processing	2.95 ± 0.3 b	3.74 ± 0.39 b	3.38 ± 0.27 b
	Deep learning	0.33 ± 0.0 c	0.33 ± 0.0 c	0.33 ± 0.0 c
	p-value	***	***	***
Velvetleaf	Manual	5.76 ± 0.48 a	50.42 ± 2.22 a	71.82 ± 1.02 a
	Image processing	5.9 ± 0.41 a	2.95 ± 0.28 b	2.51 ± 0.13 b
	Deep learning	0.32 ± 0.0 b	0.33 ± 0.0 c	0.34 ± 0.0 c
	p-value	***	***	***
Wheat	Manual	7.83 ± 0.23 a	48.95 ± 1.8 a	68.39 ± 1.13 a
	Image processing	5.03 ± 0.31 b	2.28 ± 0.19 b	1.94 ± 0.25 b
	Deep learning	0.33 ± 0.0 c	0.34 ± 0.0 c	0.35 ± 0.0 c
	p-value	***	***	***
Zoysiagrass	Manual	4.97 ± 0.2 a	37.7 ± 1.54	50.83 ± 1.47 a
	Image processing	3.18 ± 0.35 b	3.65 ± 0.25 b	3.36 ± 0.38 b
	Deep learning	0.33 ± 0.0 c	0.33 ± 0.0 c	0.34 ± 0.0 c
	p-value	***	***	***

a NS, not significant; ***significant at the p-value ≤ 0.001 level.

b Ground truth seed count was manually verified seed count used for accuracy comparison.

c For the same plant species, means within the column followed by the same letter are not significantly different based on Fisher’s Protected LSD at the 0.05 probability level.

The IP-based counting method demonstrated a significant improvement in time efficiency compared to manual counting, although the time required varied by species and seed quantity. For instance, counting 70 barnyardgrass seeds took 4.0 (± 0.21) seconds, while counting 100 seeds took 5.92 (± 0.42) seconds. Notably, IP-based counting performed particularly well for species such as wheat, where counting 100 seeds required only 1.94 (± 0.25) seconds. Statistical analysis revealed that IP-based counting was significantly faster than manual counting (p< 0.05) in most cases, making it a more feasible option for moderately high-throughput scenarios.

The DL-based counting was consistently the fastest method, maintaining an average counting time of around 0.33 seconds across all species and seed quantities, demonstrating exceptional efficiency. This minimal time variance, even at higher seed counts, highlights DL’s robustness and scalability for large-scale seed-counting apps. Statistical analysis further confirmed that DL was significantly faster than both manual and IP-based counting methods (p< 0.05) across all species and seed counts, demonstrating its exceptional efficiency for high-throughput apps.

## 4 Discussion

### 4.1 Accuracy

Our findings indicate that both the IP-based and DL-based counting methods performed comparably to manual counting in terms of seed count accuracy across most species. Statistical analysis confirmed no significant differences in accuracy among the three methods (p > 0.05) for most species, except for Kentucky bluegrass, redroot pigweed, and smooth pigweed.

The DL model exhibited an advanced capacity to recognize complex patterns within seed images, showing potential for distinguishing seeds from the background and separating them from one another under specific conditions. This capability was particularly advantageous in scenarios with dense clusters of seeds or where visual complexity could challenge human counters. However, this did not translate into a significant advantage in overall accuracy over manual counting.

Unexpectedly, the DL model underperformed in some high-seed-count scenarios, particularly for smaller or visually ambiguous seeds, such as smooth pigweed and Kentucky bluegrass. For instance, in a 100-seed count test for smooth pigweed, the model identified only 27.33 (± 0.42) seeds, resulting in a statistically significant undercount (p< 0.001) compared to both manual and IP-based methods. This limitation can be attributed to the inherent complexity of detecting smaller or densely clustered seeds, where visual ambiguity and overlapping shapes pose a challenge for DL models. Although the model showed promising results for other seed species, this inconsistency highlights the need for further optimization to enhance its robustness across different seed types and environmental conditions.

The IP-based method also achieved comparable accuracy to manual counting but demonstrated a dependency on specific IP conditions, particularly controlled lighting. In the present study, an acrylic sheet was used to achieve uniform lighting across images; however, this setup did not yield a noticeable improvement in seed count accuracy. Minor discrepancies were observed, with IP-based counting sometimes showing a slight tendency toward overcounting, as seen in barnyardgrass, where it counted 100.5 (± 0.22) seeds, where the actual count was 100 seeds. Despite these minor deviations, they remained statistically insignificant (p > 0.05) for most species. This suggests that IP-based counting could serve as an efficient and cost-effective alternative to manual counting without compromising accuracy, provided that environmental conditions, especially lighting, are properly controlled.

### 4.2 Time efficiency

In terms of time efficiency, both automated methods demonstrated substantial advantages over manual counting, especially as the number of seeds increased. The DL-based method was the fastest, with an average processing time of approximately 0.33 seconds per image, regardless of seed type or quantity. This efficiency was highly significant (p< 0.001) compared to manual counting, which required 76.1 (± 1.24) seconds for 100 maize seeds and over 93.59 (± 1.84) seconds for 100 smooth pigweed seeds. The minimal variance in time for DL counting across different seed quantities and types highlights its robustness and suitability for high-throughput apps, where large volumes of seeds need to be processed within a limited timeframe. Additionally, its capability to perform real-time IP without specialized equipment further enhances its potential utility in large-scale agricultural and commercial apps.

The IP-based method was also faster than manual counting but required an initial setup time due to the need for an acrylic sheet and controlled lighting conditions. For instance, IP-based method counted 100 wheat seeds in 1.94 (± 0.25) seconds and 100 barnyardgrass seeds in 5.92 (± 0.42) seconds, showing variability based on species and seed count. Although IP-based method provided significantly quicker counts than manual methods (p< 0.05), the reliance on specific setup conditions limits its scalability in field apps or variable lighting environments.

Manual counting, though accurate, proved to be the most time-intensive and laborious, with processing times increasing considerably with higher seed counts. While manageable for small quantities, its inefficiency became apparent as the number of seeds grew, underscoring its impracticality for large-scale counting purposes.

These findings emphasize the critical need for automated methods in large-scale seed counting, where time efficiency is essential for operational viability. Both the DL-based and IP-based methods offer significant improvements in time efficiency, with DL particularly standing out for its speed, scalability, and minimal setup requirements.

### 4.3 Practical implications

The integration of automated seed counting methods into a mobile app offers a practical and accessible solution for researchers and practitioners in agriculture. This mobile app enables quick and accurate seed counting in the field or laboratory, significantly reducing the time and labor required for manual counting.

The DL-based method, in particular, provides substantial advantages due to its high accuracy and user-friendly design. By eliminating the need for specialized equipment, this method is more versatile and can be readily adopted in various settings, from research laboratories to on-site field studies.

The mobile app for automatic seed counting has versatile apps, enhancing efficiency and accuracy in seed-related tasks. In research laboratories, agricultural researchers can utilize the app to swiftly and precisely count seeds in experimental studies, significantly reducing the time and labor required. During field studies, it supports real-time data collection and analysis, allowing on-site seed counting with minimal disruption to workflows.

Seed production facilities can integrate the app into their processes, ensuring accurate and efficient seed counting during production and packaging. Educational institutions can incorporate the app into their agricultural science programs, providing students with hands-on experience using advanced seed counting technology. Agricultural extension services can utilize the app to support farmers in seed counting tasks, thereby enhancing the accuracy of seed-related recommendations. Additionally, plant breeders can use the app to streamline seed counting in breeding trials, facilitating the selection of varieties with greater efficiency.

This tool offers transformative potential in optimizing seed counting across a wide range of agricultural apps, driving advancements in research, education, and industry practices. By addressing the challenges associated with manual seed counting and exploring advanced technological solutions, the developed seed counting tools illustrated in this study enhance the accuracy and efficiency of agricultural research and crop and weed management practices.

### 4.4 Limitations and future research

Although the results of this study are promising, several limitations need to be addressed. The accuracy of the DL model is influenced by the quality and diversity of the training data. As observed in the results, the model performs well on most seed species but faces challenges with seeds that have more complex visual characteristics. This inconsistency highlights the need for further optimization.

Future research will focus on improving the model’s performance in these challenging cases. The training process will be enhanced by incorporating more data augmentation techniques, such as variations in lighting, background, and seed types, to increase model robustness across different environments. In addition, future experiments should evaluate model performance directly under field conditions, incorporating real-world complexities such as natural sunlight variation, uneven surfaces, and mixed seed arrangements, which were not fully captured in the current setup. The dataset will also be expanded to include a broader range of seed species, with more variation in seed conditions, to address the model’s performance on underrepresented seed types.

Furthermore, alternative algorithms, such as contour-based detection or watershed segmentation, could be explored for specific cases where seed shapes or clustering present challenges. The integration of additional features into the mobile app is a promising avenue for future work. For instance, the ability to count seeds in mixed-species samples or differentiate between viable and non-viable seeds would significantly enhance the app’s utility. Such improvements will not only address the model’s current limitations but also enhance its adaptability to real-world agricultural scenarios, thereby increasing its relevance and practical applicability.

## 5 Conclusions

This study demonstrates that automated methods, including DL-based and IP-based approaches, offer substantial advantages over manual counting in terms of efficiency while maintaining comparable accuracy for most seed types. Manual counting, although accurate, is labor-intensive and time-consuming, making it unsuitable for large-scale apps. The DL-based method stands out for its exceptional speed and scalability, while the IP-based approach provides reliable results under controlled conditions. Both automated methods significantly reduce the time and effort required for seed counting, demonstrating their potential to optimize agricultural research and practice. Their integration into a mobile app further emphasizes their practicality, providing an accessible and efficient solution for researchers, seed producers, and agricultural professionals.

## Data Availability

The raw data supporting the conclusions of this article will be made available by the authors, without undue reservation.
